# Comprehensive analysis of endoplasmic reticulum stress and immune infiltration in major depressive disorder

**DOI:** 10.3389/fpsyt.2022.1008124

**Published:** 2022-10-24

**Authors:** Jing Zhang, Shujun Xie, Yujia Chen, Xin Zhou, Zhuanfang Zheng, Lingling Yang, Yan Li

**Affiliations:** ^1^The Second Clinical Medical College, Guangzhou University of Chinese Medicine, Guangzhou, China; ^2^Department of Internal Medicine Teaching and Research, The Third Affiliated Hospital of Guangzhou University of Chinese Medicine, Guangzhou, China; ^3^Department of Psychological Sleep, Guangdong Provincial Hospital of Chinese Medicine, Guangzhou, China

**Keywords:** endoplasmic reticulum stress-related differentially expressed genes (ERSR-DEGs), bioinformatics analysis, major depressive disorder (MDD), endoplasmic reticulum stress (ERS), immune infiltration

## Abstract

**Background:**

Major depressive disorder (MDD) is a life-threatening, debilitating mental health condition. An important factor in the development of depression is endoplasmic reticulum stress (ERS). However, their roles in MDD have not yet been established. The goal of this study was to examine ERS and its underlying molecular mechanisms in MDD.

**Methods:**

We used data from two microarray datasets (GSE98793 and GSE39653) and the GeneCards database to examine the reticulum stress-related differentially expressed genes (ERSR-DEGs) associated with MDD. Gene Ontology (GO), Kyoto Encyclopedia of Genes and Genomes (KEGG), Gene Set Enrichment Analysis (GSEA), and Gene Set Variation Analysis (GSVA) were used to further investigate the function and mechanism of ERS in MDD. Moreover, we constructed protein-protein interaction (PPI) networks to identify hub genes as well as the regulatory network of microRNAs (miRNAs), transcription factors (TFs), and potential drugs related to ERSR-DEGs. CIBERSORT was then used to evaluate the immune activity of MDD samples and conduct a correlation analysis between the hub genes and immune cells.

**Results:**

In total, 37 ERSR-DEGs and five hub genes were identified (NCF1, MAPK14, CASP1, CYBA, and TNF). Functional enrichment analysis revealed that ERSR-DEGs were predominantly enriched in inflammation-and immunity-related pathways, such as tumor necrosis factor signaling, NF-κB signaling, and Toll-like receptor signaling pathways. Additionally, 179 miRNAs, 25 TFs, and 15 potential drugs were tested for their interactions with the ERSR-DEGs. CIBERSORT found high proportions of Tregs, monocytes, and macrophages M0 in the MDD samples. Among these, hub genes showed a significant correlation with immune cell infiltration in patients with MDD.

**Conclusions:**

NCF1, MAPK14, CASP1, CYBA, and TNF are potential ERS-related biomarkers for the diagnosis of MDD. Our research has revealed a significant correlation between immune cells and ERS-related genes with MDD. Not only did our study contribute to a better understanding of the regulatory mechanisms of ERS in underlying MDD pathology, but it also established a paradigm for future studies on ERS.

## Introduction

Major depressive disorder (MDD) is a severe, recurrent, and life-threatening mental disorder with a high prevalence and low remission rate (1). According to epidemiological statistics from US adults, the lifetime prevalence of MDD is 20.6% and the 12-month prevalence is 10.4% (2). Depression seriously affects psychosocial functioning and is the primary cause of disability (3). MDD is a highly heterogeneous and multifactorial disease, making diagnosis and therapy more challenging (4). Even though various research has been undertaken to discover biomarkers for the diagnosis of MDD, most have not yet been applied in the clinical context due to insufficient specificity and efficacy (5–7). Although numerous antidepressants exist, only 30% of patients achieve complete remission, reflecting current therapies fail to address important biological processes involved in MDD pathology (8, 9). Therefore, discovering the biological basis of MDD progression and identifying novel diagnostic indicators and treatment targets for patients is crucial.

ERS, characterized by the accumulation of incompletely folded and unfolded proteins in the lumen, stimulates the unfolded protein response (UPR) (10). Numerous studies (11–13) have demonstrated that ERS is closely linked with the pathophysiology of depression. According to reports, the ERS signaling proteins GRP78, CHOP, and XBP1 are continuously activated in MDD patients (14). Moreover, a variety of pathological processes are associated with ERS, such as immunological responses, inflammatory responses, and oxidative stress (15–17), and these mechanisms also contribute to MDD (18–21). These studies demonstrate that ERS can alter the course of depression, either directly or indirectly, through its participation in some crucial biological processes. Of note, the potential of ERS as a target for novel antidepressants has been demonstrated in preclinical studies (13, 22). However, the molecular mechanisms by which ERS contributes to the pathogenesis of MDD remain unknown for the time being.

Consequently, this study aimed to investigate the association between ERS and the etiology and immunological infiltration of MDD, using an exhaustive bioinformatics approach. Two microarray datasets from patients with MDD were downloaded from the Gene Expression Omnibus (GEO) database, along with endoplasmic reticulum stress-related genes (ERGs) from the GeneCards Database. Next, reticulum stress-related differentially expressed genes (ERSR-DEGs) were screened. Based on these ERSR-DEGs, we conducted functional annotation, including Gene Ontology and Kyoto Encyclopedia of Genes and Genomes (KEGG) enrichment analyses. Statistically significant pathways related to MDD were determined by Gene Set Enrichment Analysis (GSEA) and Gene Set Variation Analysis (GSVA) for further validation. Furthermore, a protein-protein interaction network for ERSR-DEGs, as well as potential drugs and transcription factors to target ERSR-DEGs, was created. Five hub genes were identified from 37 ERSR-DEGs, and RNA binding proteins (RBPs) -hub gene networks were constructed. Using the CIBERSORT software, we also explored the correlation between hub genes and immune cell infiltration. Based on our understanding of ERS and its relation to immunological infiltrations, we may find promising biomarkers or therapeutic targets for MDD.

## Materials and methods

### Data pretreatment and identification of differentially expressed genes

We retrieved two microarray datasets from the GEO database using the GEO query R package (https://www.ncbi.nlm.nih.gov/geo/) (23, 24). The first dataset, GSE98793 (25), contains 64 samples from patients with MDD and 64 samples from healthy individuals, derived from the GPL570 sequencing platform. The second dataset, GSE39653 (26), obtained from the GPL10558 sequencing platform, included 21 blood samples from patients with MDD and 24 blood samples from healthy individuals. Table 1 lists the details of the dataset.

**Table 1:** The characteristics of the two microarray datasets.

**Dataset**	**Organism**	**Tissue**	**Array platform**	**MDD samples**	**HC samples**	**Study**
GSE98793	Homo sapiens	Whole blood samples	GPL570 [HG-U133_Plus_2] Affymetrix Human Genome U133 Plus 2.0 Array	64/128	64/128	Leday et al. (25)
GSE39653	Homo sapiens	Whole blood samples	GPL10558 Illumina HumanHT-12 V4.0 expression beadchip	21/45	24/45	Savitz et al. (26)

The GEO datasets were quantile-normalized using the normalized between arrays function (27) from the “limma” R package (28) and visualized using a boxplot. Additionally, principal components analysis (PCA) was used to visualize the discrepancy between MDD and control groups using the “factoextra” R package (29). Differentially expressed genes (DEGs) were screened utilizing the “limma” program (*P*-value < 0.05 and |logFC| > 0).

The GeneCards (https://www.genecards.org/) database is a searchable, integrated resource for comprehensive, user-friendly, annotated, and predicted information (30). We retrieved ERGs with a relevance score >2 from GeneCards searched using the keyword “Endoplasmic Reticulum Stress.” We identified ERSR-DEGs by intersecting ERGs with DEGs in GSE98793 and GSE39653. The expression levels of ERSR-DEGs in the GSE98793 and GSE39653 datasets were visualized using the R package “heatmap”.

### GO and KEGG enrichment analysis

To obtain a better understanding of the ERSR-DEGs, GO annotation, and KEGG pathway enrichment analyses were performed. Bioconductor's “clusterProfiler” (31) and “pathview” (32) packages in the R software were utilized for GO enrichment, KEGG pathway analysis, and plotting. Benjamin-Hochberg (BH) adjustment was used to calculate the false discovery rate (FDR). A q-value of 0.05 was used as the cut-off criterion.

### Gene set enrichment analysis and gene set variation analysis

Gene set enrichment analysis (GSEA) is used to analyze the contribution of individual genes to phenotype by assessing the distribution of preset gene sets across gene lists sorted by phenotype correlation (33). GSVA is a non-parametric and unsupervised method for estimating the score of a pathway or signature based on transcriptome data (34). We used GSEA on the GSE98793 dataset to analyze global patterns of differential gene expression and to determine if there were gene expression features that were significantly enriched in either the MDD or normal groups. GSEA analyses were conducted using the reference gene sets “h.all.v7.2. symbols.gmt”, “c2.kegg.v7.4. symbols” and “c5.go.v7.4. symbols” in the MSigDB database (35). Using the same dataset, GSVA was employed to compare pathway enrichment in the MDD and control groups.

### PPI network construction and identification of the hub genes

GeneMANIA (36) and STRING (37) are two wellknown web tools that provide a list of genes associated with a query gene based on diverse biological associations. An investigation of the association between genes can clarify the role of genes in biological processes. We analyzed the PPI network of ERSR-DEGs using GeneMANIA software and the STRING database, followed by visualization using Cytoscape (38). Meanwhile, utilizing the CytoHubba (39) plug-in and MCC algorithm, the top five hub genes were selected.

### Construction of the molecule-molecule network

RNA-binding proteins are thought to be critical for gene regulation through the regulation of RNA stability and translation. In certain diseases, miRNAs and transcription factors interact with target genes to regulate gene expression (40). We identified RBPs that target hub genes using ENCORI (https://starbase.sysu.edu.cn/) (41). MiRNAs associated with ERSR-DEGs were identified using the ENCORI (41), TargetScan (42), and miRDB databases (43). Significant transcription factors (TFs) were identified using the JASPAR database (44).

DSigDB is a comprehensive database for identifying targeted drugs related to DEGs (45). The database has 22 527 gene sets and is an accessible way to access disease or drug functions through the Enrichr website (46). In this study, the prediction of protein-drug interactions or molecular drug identifications is a crucial component. DSigDB was used to search for drugs or chemicals that might interact with the ERSR-DEGs.

### Evaluation of immune cell infiltration

Based on the principle of linear support vector regression, CIBERSORT is used to deconvolve the expression matrices of immune cell subgroups (47). We applied CIBERSORT to assess the proportions of immune cell types in the MDD and healthy control groups. Spearman's correlation was used to analyze the relationship between hub genes and immune cells in patients with MDD.

### Statistical analysis

The R software was used for all data processing and analyses (version 4.0.2). For the comparison of two sets of continuous variables, the Student's *t*-test was used to analyze the statistical significance of normally distributed variables, while the Mann-Whitney U test was used for variables that were not normally distributed. In this study, all statistical *p*-values were two-sided and *p* < 0.05 was considered statistically significant.

## Results

### Identification of ERSR-DEGs

**Figure 1:**
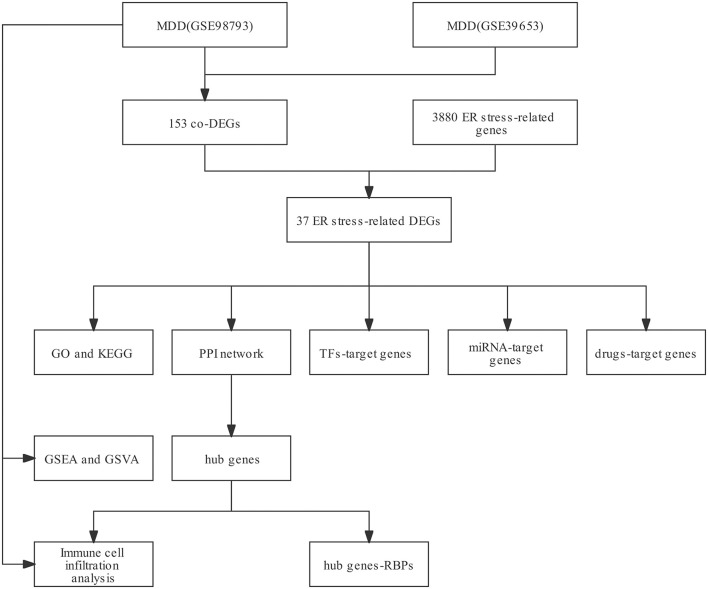
The workflow of this study.

**Figure 2:**
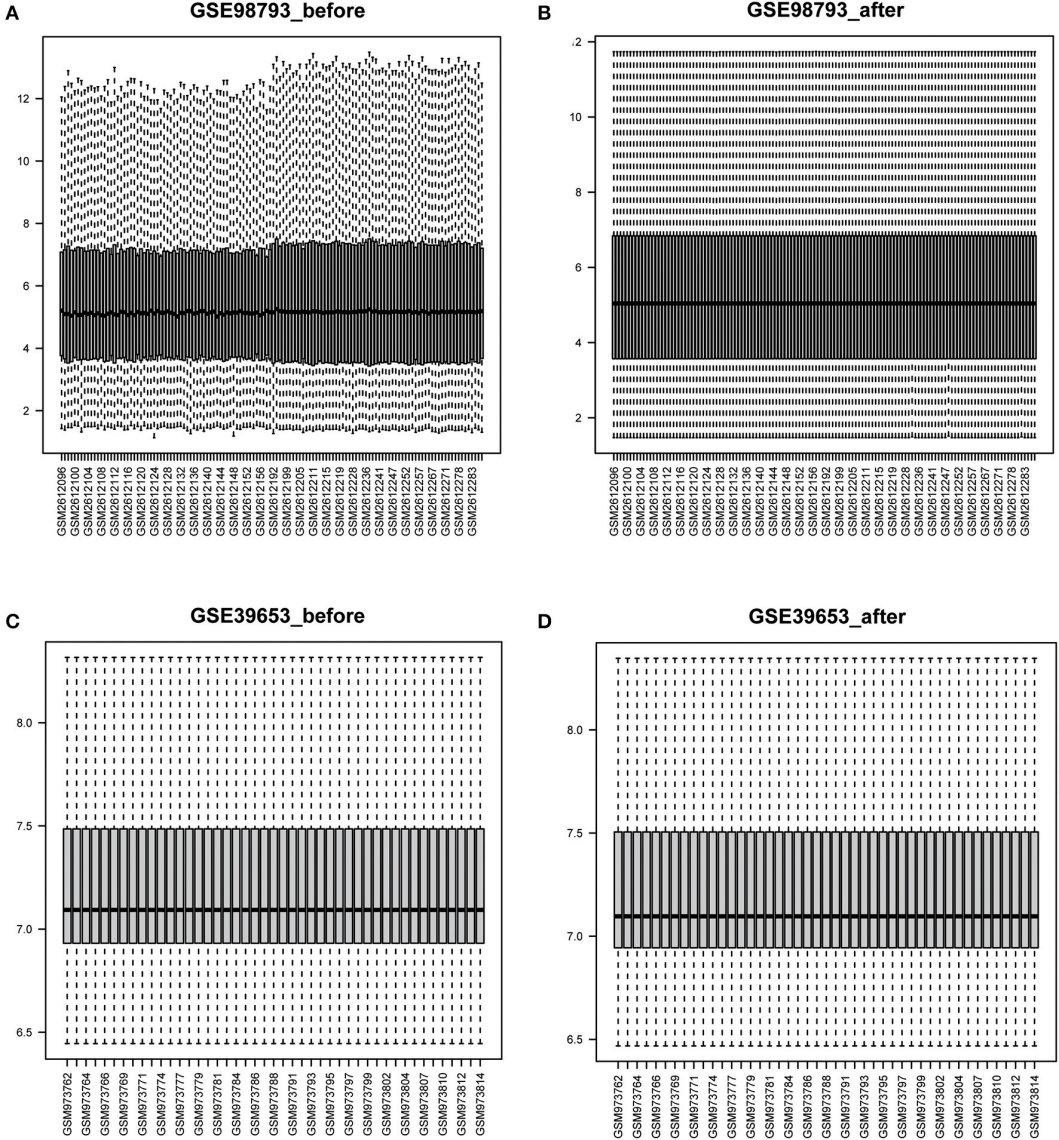
Boxplots of the gene expression data before and after normalization. **(A)** The boxplot of GSE98793 data before normalization. **(B)** The boxplot of GSE98793 data after normalization. **(C)** The boxplot of GSE39653 data before normalization. **(D)** The boxplot of GSE39653 data after normalization.

**Figure 3:**
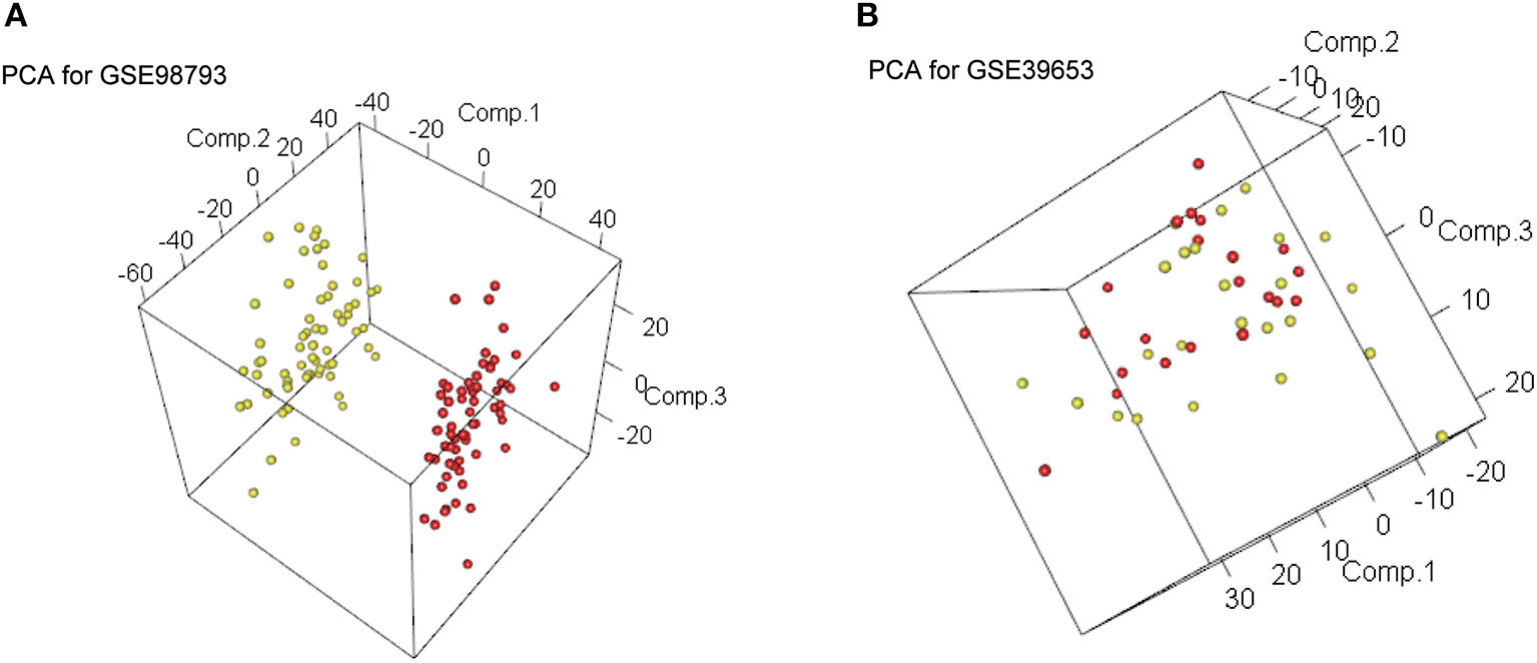
Principal component analysis (PCA). **(A)** PCA plot for GSE98793. **(B)** PCA plot for GSE39653.

**Figure 4:**
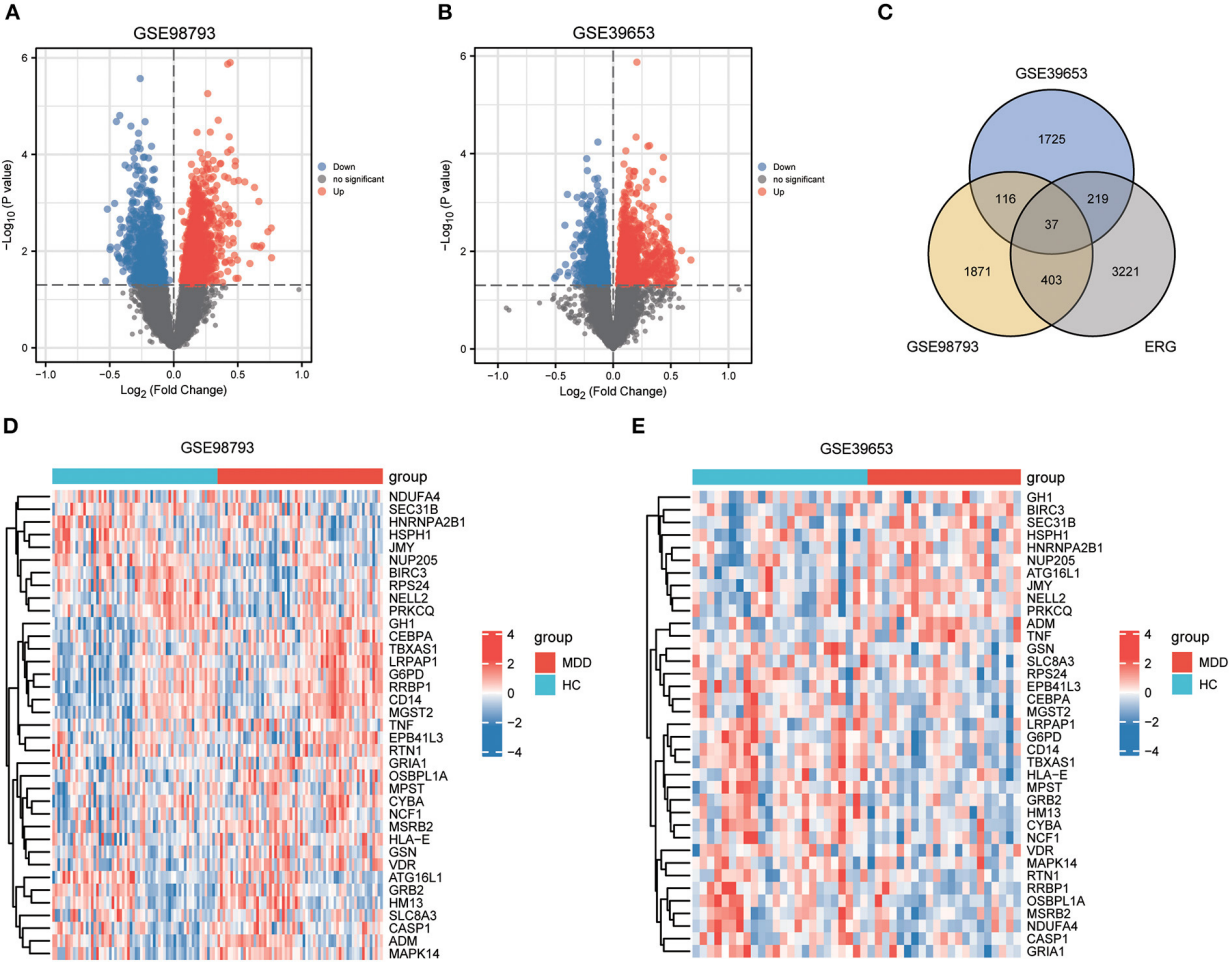
Endoplasmic reticulum stress-related gene (ERSR-DEGs) expressions. **(A)** Volcano plot of GSE98793. **(B)** Volcano plot of GSE39653. **(C)** Venn diagram for overlapping ERSR-DEGs based on 3 datasets. **(D)** Heatmap of ERSR-DEGs identified in GSE98793. **(E)** Heatmap of ERSR-DEGs identified in GSE39653.

The workflow of this study is illustrated in Figure 1. After standardizing the datasets (Figure 2), we detected DEGs between the MDD and healthy control groups. In total, 2,427 and 2,097 DEGs were extracted from GSE98793 and GSE39653, respectively, according to the predefined criteria. In addition, the separation between the MDD and control samples was excellent in the PCA analysis of the GSE98793 and GSE39653 datasets (Figure 3). Among the DEGs depicted in the volcano plots, GSE98793 contained 1,472 upregulated genes and 955 downregulated genes, whereas GSE39653 contained 1,128 upregulated genes and 969 downregulated genes (Figures 4A,B). A total of 153 common differentially expressed genes (co-DEGs) were identified from the two datasets. In addition, 3,880 ERGs were collected from GeneCards using the specified criteria (Supplementary Table 3). The co-expressed ERSR-DEGs were integrated using a Venn diagram in R (Figure 4C). A total of 37 ERSR-DEGs were extracted, and the expression levels of these genes in GSE98793 and GSE39653 were further visualized using a heat map (Figures 4D,E).

### Gene ontology and KEGG pathway analysis

**Figure 5:**
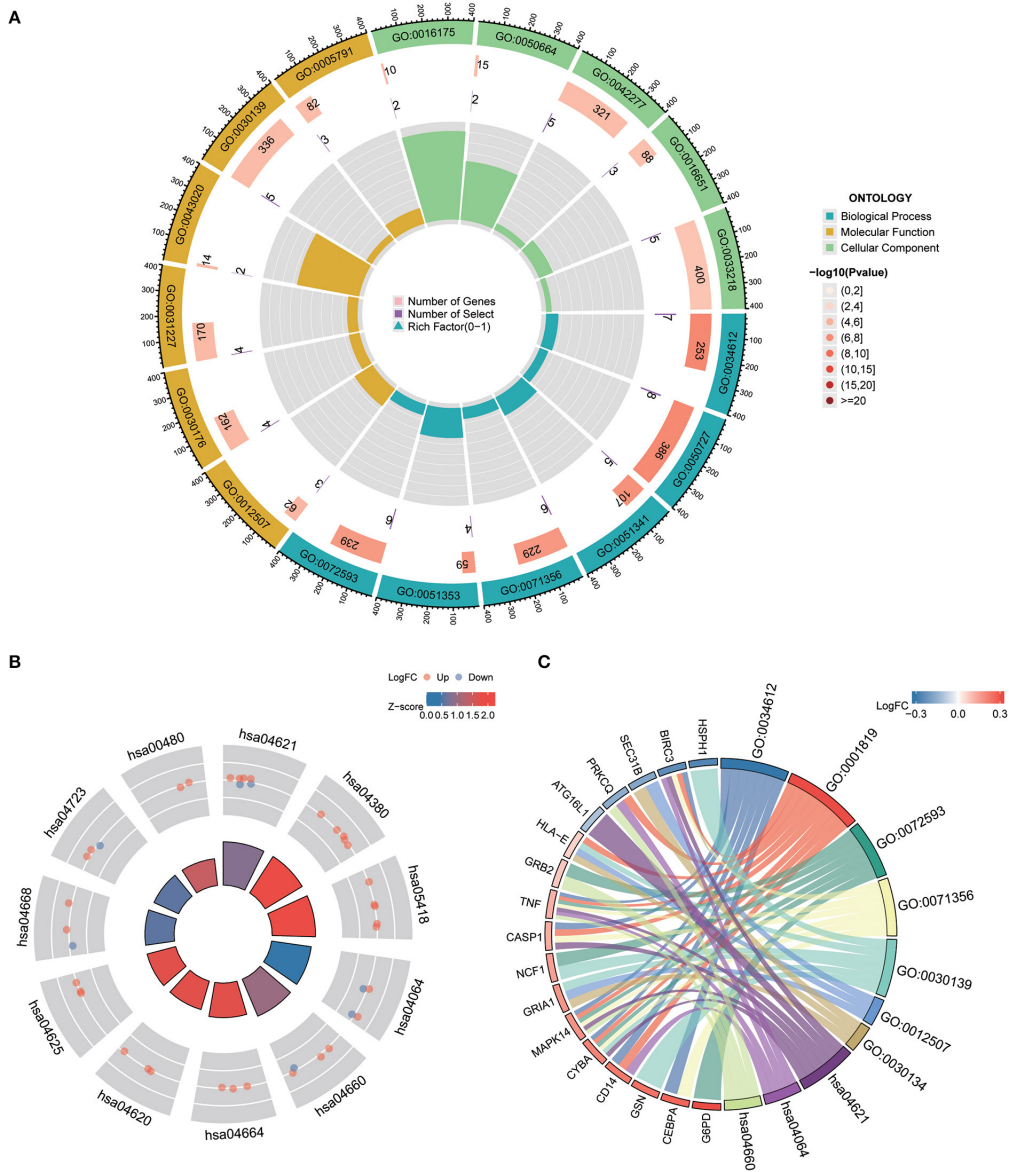
GO functional and KEGG pathway enrichment analysis of ERSR-DEGs. **(A)** GO enrichment analysis of ERSR-DEGs. **(B)** KEGG pathway analysis of ERSR-DEGs. Red nodes suggest upregulated genes, and blue nodes mean downregulated genes. Quadrilateral color indicates Z-score. Red indicates a positive Z-score (activated function), and blue indicates a negative Z-score (inhibited function). **(C)** The chord plot displays the relationship between ERSR-DEGs and GO and KEGG entries.

To understand the functional properties of ERSR-DEGs, GO enrichment and KEGG pathway analyses were conducted (Figure 5; Table 2). The GO enrichment results showed that for BP, ERSR-DEGs were considerably enriched in response to the tumor necrosis factor, inflammatory response regulation, oxidoreductase activity regulation, etc. For CC, ERSR-DEGs were primarily abundant in the ER to Golgi transport vesicle membrane, NADPH oxidase complex, an integral component of the endoplasmic reticulum membrane, and rough endoplasmic reticulum. In MF, ERSR-DEGs were markedly enriched in superoxide-generating NADPH oxidase activity, oxidoreductase activity, and NADPH.

**Table 2:** The details of GO and KEGG terms for ERSR-DEGs with their corresponding *p*-values.

**Gene ontology**	**ID**	**Description**	***p*-value**	**Count**
BP	GO:0034612	Response to tumor necrosis factor	5.52E-07	7
BP	GO:0050727	Regulation of inflammatory response	6.95E-07	8
BP	GO:0051341	Regulation of oxidoreductase activity	2.09E-06	5
BP	GO:0071356	Cellular response to tumor necrosis factor	5.31E-06	6
BP	GO:0051353	Positive regulation of oxidoreductase activity	5.43E-06	4
BP	GO:0072593	Reactive oxygen species metabolic process	6.78E-06	6
CC	GO:0012507	ER to Golgi transport vesicle membrane	0.000219	3
CC	GO:0030176	integral component of endoplasmic reticulum membrane	0.000242	4
CC	GO:0031227	Intrinsic component of endoplasmic reticulum membrane	0.000291	4
CC	GO:0043020	NADPH oxidase complex	0.000313	2
CC	GO:0030139	Endocytic vesicle	0.000404	5
CC	GO:0005791	rough endoplasmic reticulum	0.000499	3
MF	GO:0016175	Superoxide-generating NAD(P)H oxidase activity	0.000176	2
MF	GO:0050664	Oxidoreductase activity, acting on NAD(P)H, oxygen as acceptor	0.000408	2
MF	GO:0042277	Peptide binding	0.000435	5
MF	GO:0016651	Oxidoreductase activity, acting on NAD(P)H	0.000734	3
MF	GO:0033218	Amide binding	0.001173	5
KEGG	hsa04621	NOD-like receptor signaling pathway	4.43E-05	6
KEGG	hsa04380	Osteoclast differentiation	9.59E-05	5
KEGG	hsa05418	Fluid shear stress and atherosclerosis	0.000142	5
KEGG	hsa04064	NF-kappa B signaling pathway	0.00055	4
KEGG	hsa04660	*T*-cell receptor signaling pathway	0.00055	4
KEGG	hsa04664	Fc epsilon RI signaling pathway	0.00197	3
KEGG	hsa04620	Toll-like receptor signaling pathway	0.006544	3
KEGG	hsa04625	C-type lectin receptor signaling pathway	0.006544	3
KEGG	hsa04668	TNF signaling pathway	0.008028	3
KEGG	hsa04723	Retrograde endocannabinoid signaling	0.017051	3
KEGG	hsa00480	Glutathione metabolism	0.01876	2

KEGG pathway enrichment analysis revealed that ERSR-DEGs were significantly enriched in immune-related pathways, including the NF-κB signaling pathway, osteoclast differentiation, NOD-like receptor (NLR) signaling pathway, Fc epsilon RI signaling circuit, Toll-like receptor (TLR) signaling pathway, and TNF signaling pathway.

**Figure 6:**
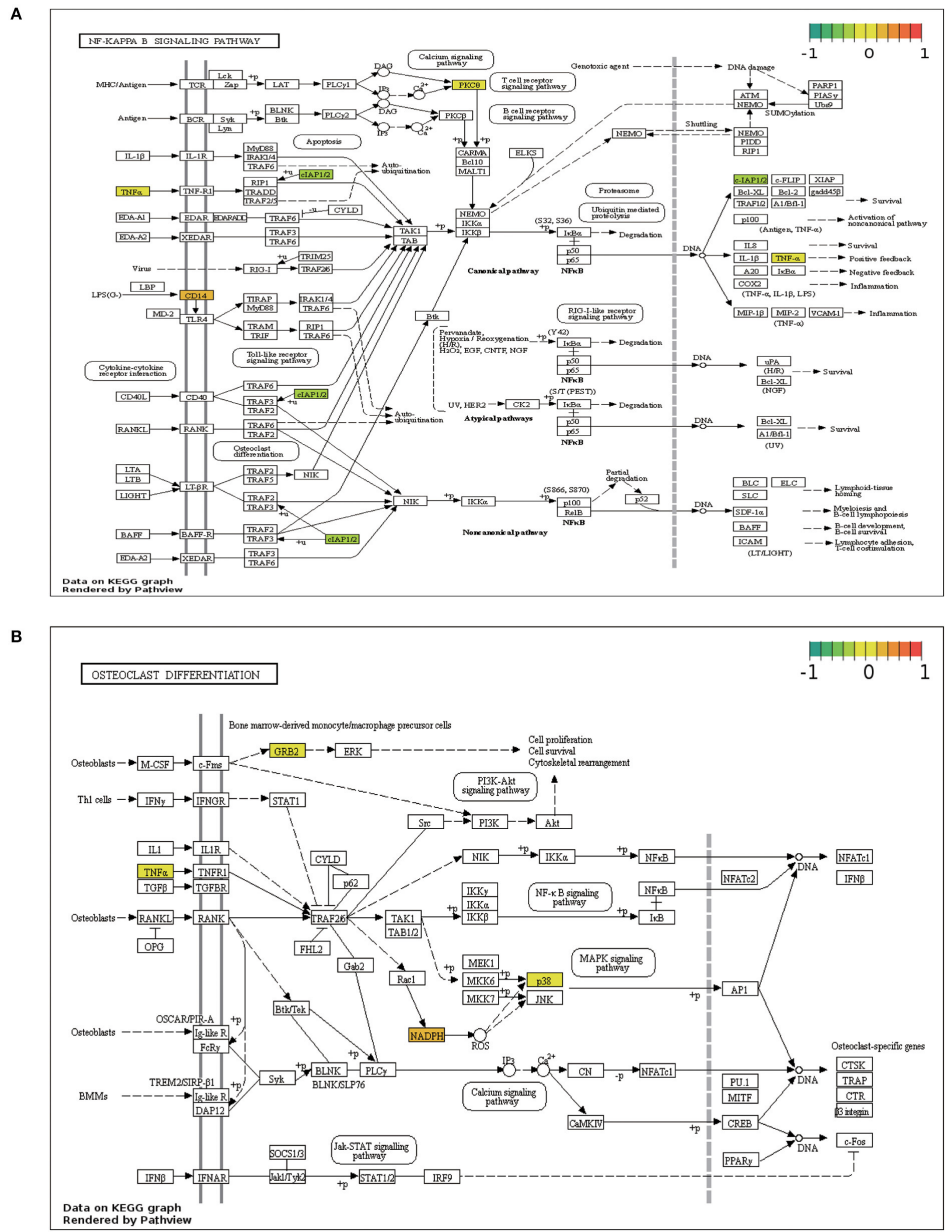
Pathview map. **(A)** NF-κB signaling pathway. **(B)** Osteoclast differentiation signaling pathway.

NF-κB signaling and osteoclast differentiation, the two strongly enriched pathways, were visualized using the Pathview program based on KEGG pathways (Figure 6).

### Gene set enrichment analysis and gene set variation analysis

**Figure 7:**
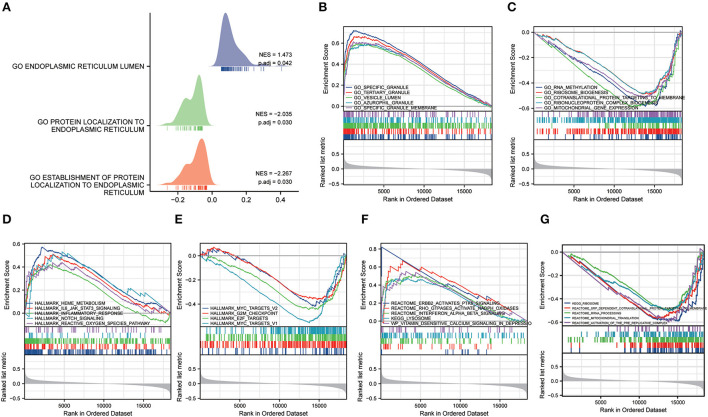
Gene set enrichment analysis (GSEA). **(A)** The ridgeline plot of the GSEA illustrates the biological processes associated with endoplasmic reticulum stress in MDD. **(B,C)** The results of GSEA (GO terms). **(D,E)** The results of GSEA (Hallmark pathways). **(F,G)** The results of GSEA (KEGG pathways).

GSEA analysis was conducted to further study the probable biological pathways and processes between the function of differential genes in MDD and healthy controls (HC), and ERS (Supplementary Table 1). The MDD group was considerably enriched in biological processes associated with ERS (Figure 7A). We discovered that the GSE98793 dataset was predominantly involved in specific granules, vesicle lumen, specific granule membrane, ran methylation, and ribosome biogenesis processes (Figures 7B,C). In addition, the highly enriched pathways in the hallmark gene sets were the IL6-JAK-STAT3 signaling pathway, the reactive oxygen species (ROS) pathway, notch signaling, E2F targets, MYC targets V2, MYC targets v1, and epithelial-mesenchymal transition (EMT) (Figures 7D,E). Among Reactome and KEGG pathways, the most enriched pathways converge on ErbB2 activate PTK6 signaling, interferon-alpha/beta (IFN-α/β) signaling, and lysosomes (Figures 7F,G).

**Figure 8:**
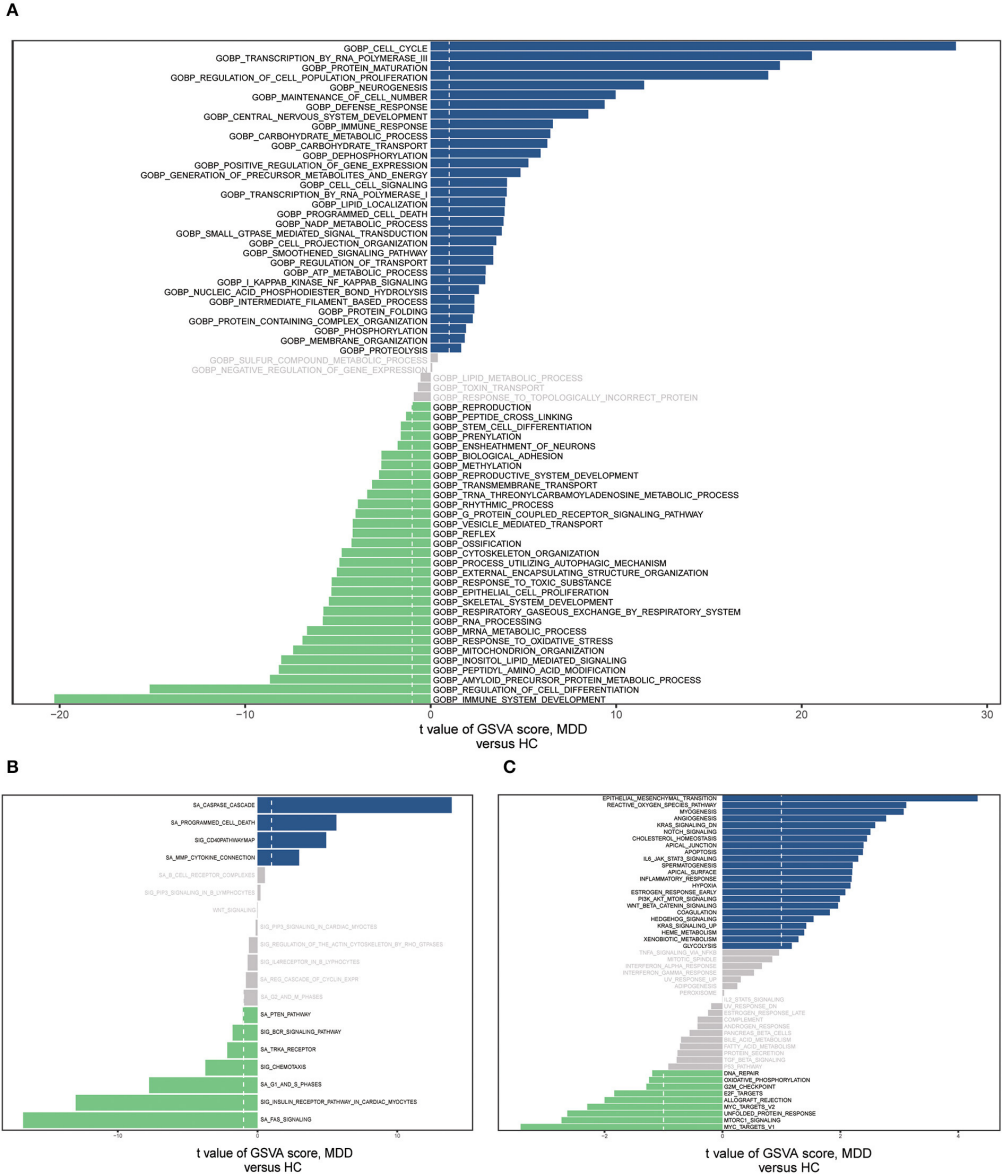
The gene set variation analysis (GSVA) for two groups based on the GSE98793 database. **(A)** Bar graph depicting “GO” pathway scores computed by the GSVA. **(B)** Bar plot of “KEGG” pathway score calculated by GSVA. **(C)** Bar plot of “Hallmark” pathway score calculated by GSVA.

We conducted GSVA analyses of the hallmark pathways, KEGG, and GO, to further investigate the biological roles of the MDD genes (Supplementary Table 2). GO analysis revealed that the MDD group genes were considerably abundant in the BP, encompassing the cell cycle, RNA polymerase III transcription, immune system development, protein maturation, and cell population proliferation regulation (Figure 8A). MDD was enriched in the following KEGG pathways: Fas signaling pathway, caspase cascade, G1 and S phases, programmed cell death, and CD40 pathway map (Figure 8B). In the hallmark gene sets, the pathways with the highest enrichment were the epithelial-mesenchymal transition (EMT), reactive oxygen species (ROS) pathway, and mTORC1 signaling (Figure 8C). According to these data, immune, inflammatory, oxidative, and apoptotic pathways, as well as biological processes related to ERS, are significantly linked to major depressive disorders.

### PPI network construction and hub genes screening

**Figure 9:**
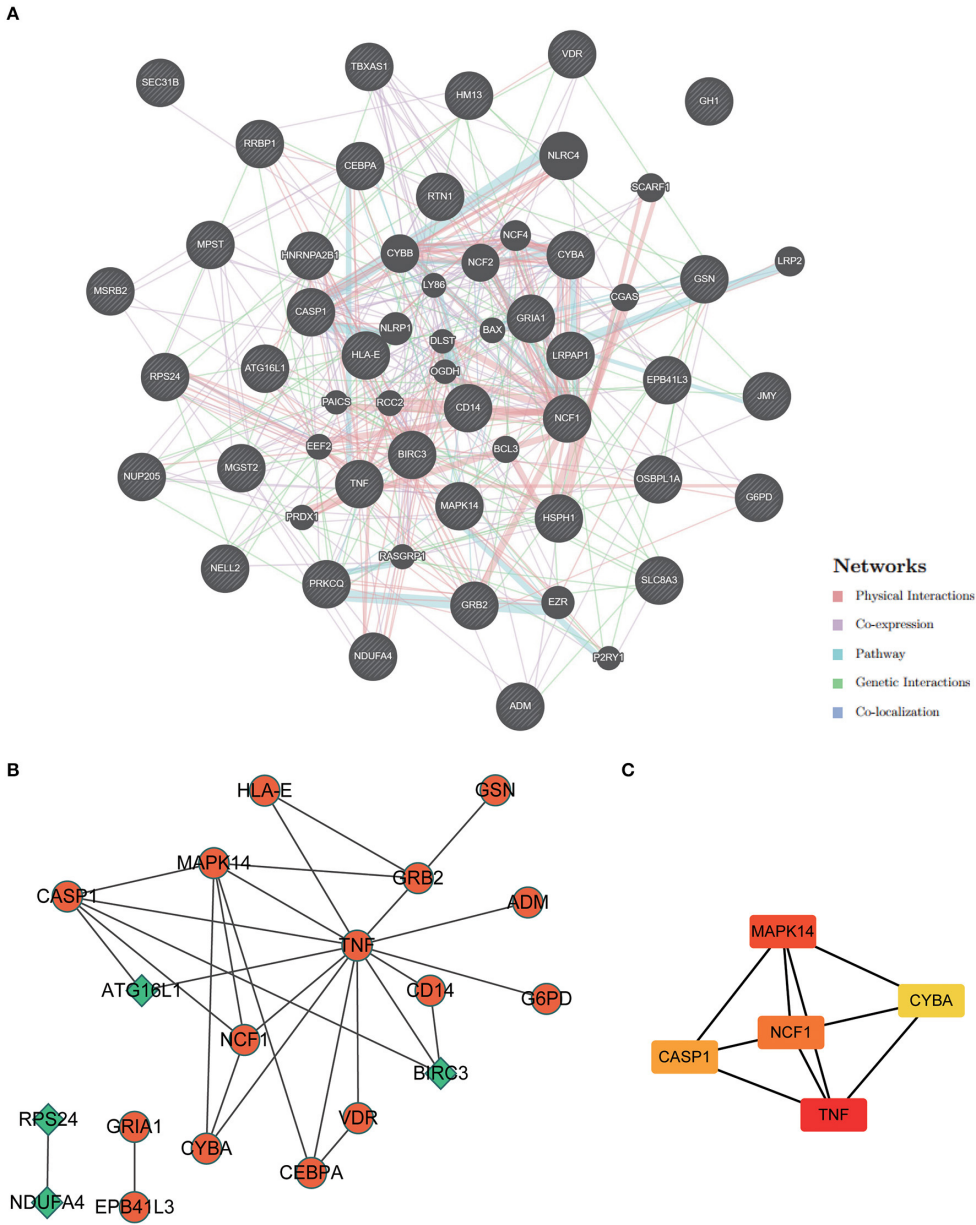
Protein-protein interaction (PPI) network analysis and hub gene screening. **(A)** The Gene interaction network of ERSR-DEGs was constructed and visualized using geneMANIA. **(B)** PPI network of ERSR-DEGs was constructed using STRING and visualized in Cytoscape. Green or orange are gene expressions down or upregulated, respectively. **(C)** Top 5 hub genes screened by MCC of cytohubba. The deeper the color, the higher the MCC score.

An analysis of GeneMANIA gene interaction networks was conducted to further investigate 37 ERSR-DEG interactions (Figure 9A). A protein-protein interaction (PPI) network of ERSR-DEGs was created using the Cytoscape program and STRING database (Figure 9B). The MCC approach using the Cytohubba plug-in selected NCF1, MAPK14, CASP1, CYBA, and TNF as the top five hub genes (Figure 9C).

### Construction of the molecule-molecule network

**Figure 10:**
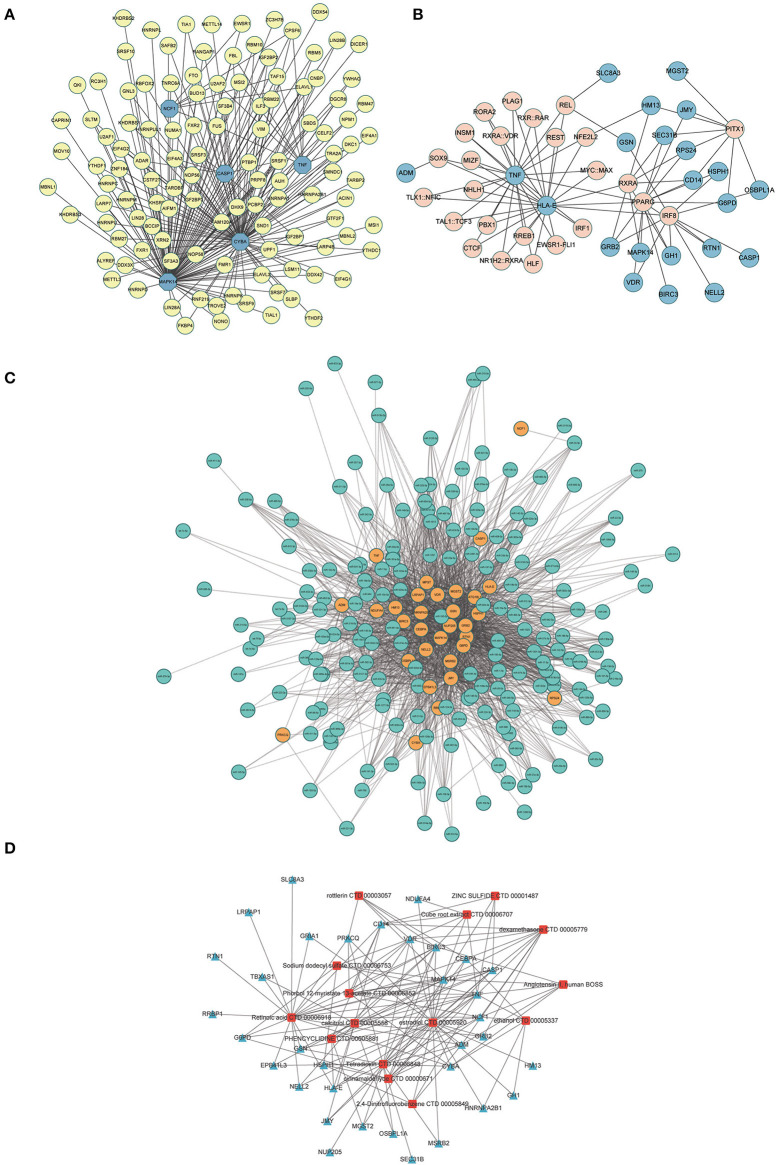
Interaction network analysis of endoplasmic reticulum stress-related differentially expressed genes (ERSR-DEGs). **(A)** In the RBPs-hub genes regulatory network, where the highlighted blue circle nodes represent the hub genes, and the yellow nodes represent RBPs. **(B)** In the TF-gene regulatory network of ERSR-DEGs, the highlighted blue circle nodes represent ERSR-DEGs, and pink nodes represent TF genes. **(C)** In the miRNA -gene regulatory network of ERSR-DEGs, the highlighted orange circle nodes represent the ERSR-DEGs, and the green nodes represent miRNA genes. **(D)** In the Drug-gene interaction network of ERSR-DEGs, where the highlighted blue squares represent the ERSR-DEGs, and the red squares represent the related drugs.

A total of 116 RBPs that bound to five hub genes were predicted using the ENCORE database (Figure 10A). To better investigate the molecular mechanisms of the 37 ERSR-DEGs in MDD, we constructed the ERSR-DEGs-TF, ERSR-DEGs-miRNA, and ERSR-DEG-Drugs regulatory networks. A regulatory network of the mRNA-TF-target gene was established, involving 22 mRNAs and 25 TFs (Figure 10B). The mRNA–miRNA interaction network contained 31 mRNAs and 179 miRNAs (Figure 10C).

Using DSigDB to predict drugs target the 37 ERSR-DEGs, 15 potential drugs or compounds for MDD were identified, including cinnamaldehyde, dexamethasone, estradiol, phencyclidine, sodium dodecyl sulfate, rottlerin, retinoic acid, calcitriol, phorbol 12-myristate 13-acetate, angiotensin II, tetra dioxin, cube root extract, zinc sulfide, 2,4-Dinitrofluorobenzene, ethanol (Figure 10D).

### Immune infiltration analysis

**Figure 11:**
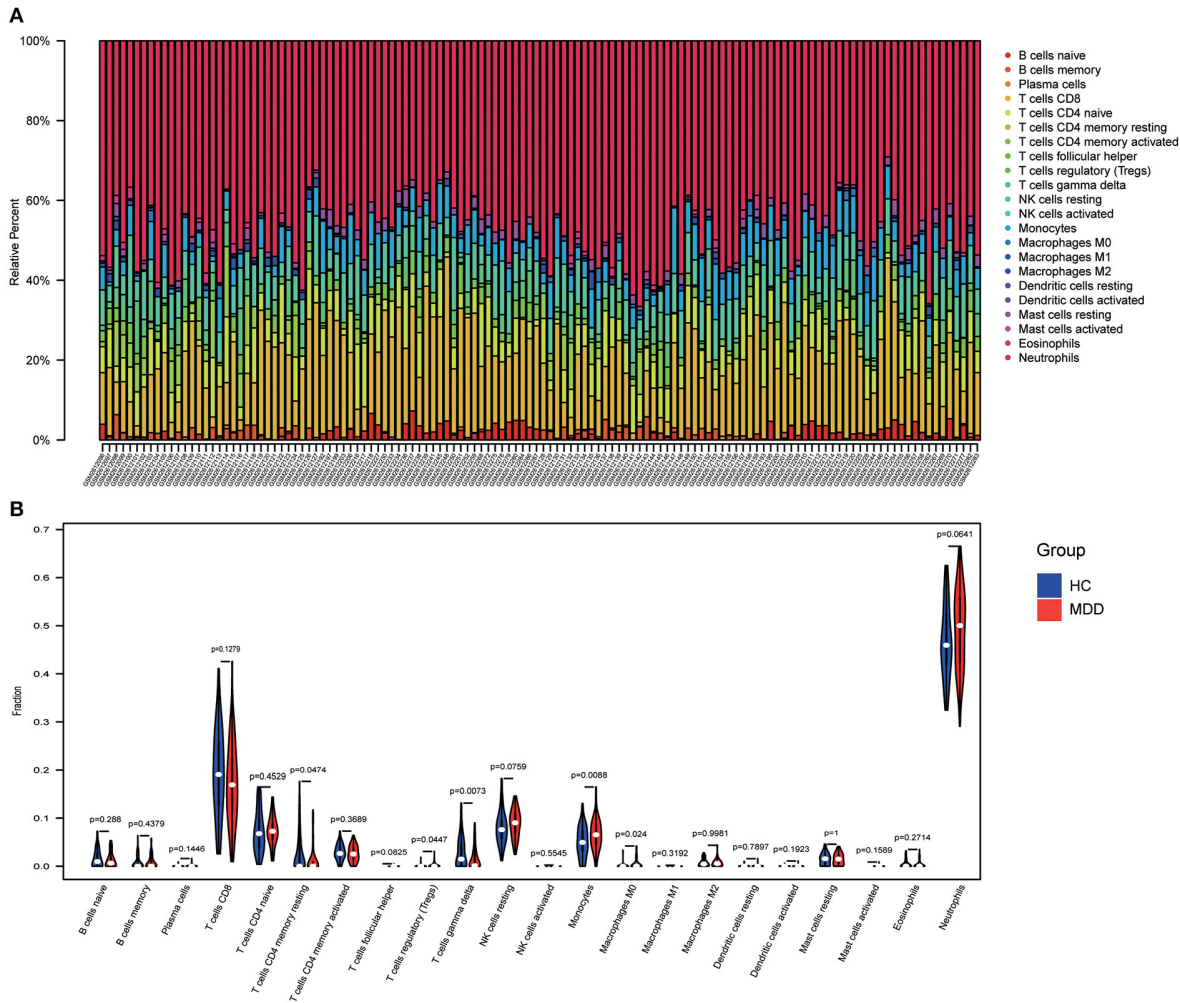
Immune infiltration analysis. **(A)** A bar chart showing the proportions of 22 different types of immunocytes in the GSE98793 dataset. **(B)** The violin plot shows the difference in immune infiltration between healthy control (HC) and MDD samples.

**Figure 12:**
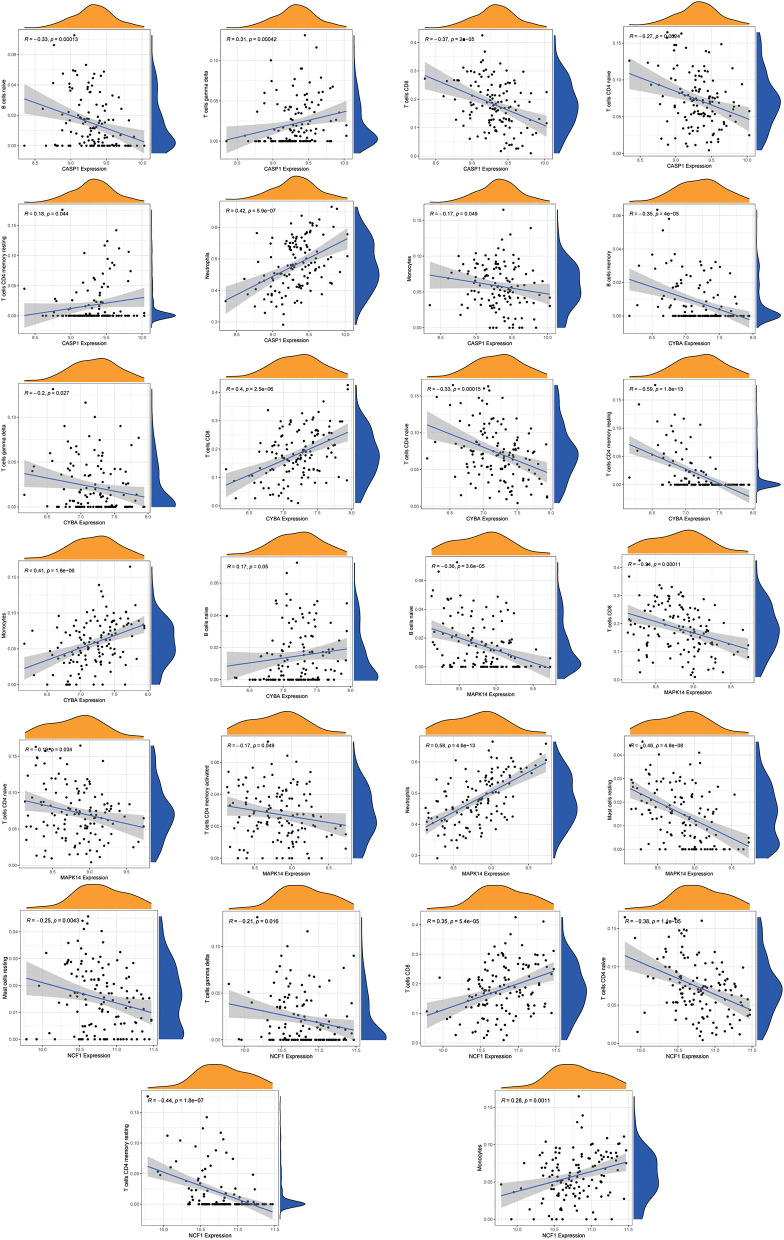
Correlation analysis of immune cell infiltration and the hub genes. The vertical axis represents the Hub genes expression level, and the horizontal axis represents infiltrated immunocytes expression level.

Using the “CIBERSORT” algorithm, the difference in immune infiltration between the MDD and HC samples of GSE98793 was estimated. The percentages of 22 specific immune cells are depicted in a bar graph using various colors for each sample (Figure 11A). Immune cell infiltration investigations demonstrated that regulatory T cells (Tregs), monocytes, and Macrophages M0 were higher in the MDD group, whereas resting T cell CD4 memory and T cell gamma delta levels were lower (Figure 11B). A scatter plot illustrates the relationship between hub gene expression and immune cell infiltration. As shown in Figure 12, correlation analysis revealed a positive correlation between CASP1 and neutrophils (R = 0.42, *P* = 5.9e-07). A positive correlation was observed between CYBA and monocyte levels (R = 0.41, *P* = 1.8e-06). MAPK14 was positively correlated with neutrophils (R = 0.58, *P* = 4.6e-13) and negatively correlated with resting mast cells (R = −0.46, *P* = 4.8e-08). A negative correlation was observed between NCF1 and CD4 memory resting T cells (R = −0.44, *P* = 1.8e-07).

## Discussion

MDD is among the most prevalent and debilitating mental disorders and has high mortality and morbidity rates (1, 2). Depression affects millions of people worldwide, placing a burden on families and communities (3). Owing to its high heterogeneity and complex pathophysiology, the identification and diagnosis of MDD remains a global problem with enormous dimensions (48). Despite extensive research efforts, MDD is yet to be definitively diagnosed using specific and sensitive biomarkers. Therefore, innovative biomarkers are required for the accurate diagnosis and treatment of MDD. Prior clinical and experimental research has demonstrated that endoplasmic reticulum stress contributes to the pathogenesis of depression (11, 13). However, little research has investigated ERS-related biomarkers of MDD and the relationship of ERS-related genes with immune infiltration in MDD. In this work, for the first time, the biological significance of ERS-related genes and their association with immune infiltration in MDD were explored in depth, and prospective biomarkers were identified.

In this study, MDD-related and ERS-related genes were identified using the GEO and GeneCards databases, respectively. After the analysis, 37 ERSR-DEGs were identified. To further understand the role of ERSR-DEGs in MDD, several functional enrichment analyses were conducted. BP analyses of the GO annotation revealed that ERSR-DEGs are involved in tumor necrosis factor response, oxidoreductase regulation, and inflammation. Among the MF annotations, NADPH oxidase activity was the most significant GO term. Consistent with our data, previous research has confirmed that NADPH oxidase (NOX) plays a critical role in promoting and maintaining depressive behavior (49). NOX is a key factor linking oxidative stress and ESR-induced apoptosis (50). KEGG analysis showed that ERSR-DEGs were mainly involved in various inflammatory signaling pathways, including the NF-κB, NLR, TLR, and TNF signaling pathways, which have collectively been confirmed as essential mechanisms in the development of MDD (51–53). In addition, GSEA and GSVA analyses revealed that most genes in the MDD group were associated with inflammation and oxidative stress-related pathways, including the IL6-JAK-STAT3 signaling pathway and the reactive oxygen species (ROS) pathway. A growing body of evidence suggests that ERS triggers inflammatory signaling pathways by interacting with UPR components and cytokine-regulating transcription factors such as TLRs, NLRs, NF-κB, and TNF-α (52, 54, 55). Among these, TLRs, which are key players in modulating inflammation and host immunity, have been shown to provide a link between depression and UPR (52). According to these findings, the interactions between ERS, inflammation, and oxidative stress may contribute to the pathogenesis of depression.

Interestingly, we found that ERSR-DEGs were highly enriched in the osteoclast differentiation pathway. A similar study found that osteoclast differentiation was significantly upregulated in bipolar disorder type II (BDII) (56). Studies have found that ERS-related PERK and CREBH pathways play crucial roles in osteoclast differentiation and function (57–59). MDD has been identified as a risk factor for low bone mineral density in previous studies (60, 61). The association between ERS and MDD as well as osteoclast differentiation has been studied separately, but their interrelationships remain unknown.

Five hub genes were identified by PPI network analysis of ERSR-DEGs: NCF1, MAPK14, CASP1, CYBA, and TNF. Recent research has revealed that the CASP1-mediated signaling pathway links environmental stress to depression-like behaviors by controlling the membrane integrity of glutamate receptors (62). CASP1 activation is a crucial choke point for inducing NLRP3 inflammasome activation in the inflammatory cascade (63). ERS has been reported to induce NLRP3 inflammasome activation in different cell types, and the interaction between ERS, NLRP3 inflammasome, and inflammation promotes the development of depression (64, 65). TNF-α has been implicated in the pathophysiology of depressive disorders and the mechanism of antidepressant treatment (51). Previous studies have shown that TNF-α can selectively activate one or more ERS pathways, triggering inflammatory or apoptotic responses (55, 66). MAPK14 (P38α) is one of the major isoforms of p38 MAPK and is activated by numerous stress conditions, including ERS and inflammatory cytokines (67). Recent research has indicated that p38 MAPK is a critical modulator of stress-induced depression-like and drug-seeking behaviors, and selective p38 MAPK deletion in serotonergic neurons promotes stress resilience (68).

For the first time, we discovered that NCF1 and CYBA might be key target genes that explain the role of ERS in the pathogenesis of depression. NCF1 (p47phox) and CYBA (p22phox) are both essential components of the NADPH oxidase system (69, 70). Pieces of evidence supported the view that NADPH oxidase plays a pivotal role in developing and maintaining depression (49, 71). Yi et al. discovered that p47phox phosphorylation is a key regulator of the signaling cascade that governs the induction of long-term depression (LTD) and synapse weakening (72). Similarly, it has been reported that heterozygous deletion of p47phox can alleviate depressed behaviors (49). It is well known that NADPH oxidase is a major source of reactive oxygen species (ROS). The NADPH oxidase subunits NOX4 and NOX2, both of which rely on p22phox, have been shown to contribute to ROS generation in response to ERS. p22phox-dependent NADPH oxidases are important mediators of ERS driving the UPR (73). Furthermore, NOX2, NOX4, and p22phox are essential signaling components in ERS-induced apoptosis (50, 73). Although there is no direct evidence of CYBA involvement in MDD, it seems likely that CYBA is acting through NADPH oxidase and ERS. Recent research has revealed that NCF1 and NOX2 complex-derived ROS are critical regulators of the immune and inflammatory pathways (70). Oxidative stress and proinflammatory signaling have been identified as contributing factors to MDD (74). Consequently, it can be speculated that ERS may contribute to depression *via* a variety of mechanisms.

Accumulating evidence has demonstrated the significance of an abnormal inflammatory response in the development of MDD (18, 75). As previously reported, the proportions of Tregs, monocytes, and M0 macrophages in patients with MDD were significantly higher than those in controls, which is consistent with our findings (76–78). In addition, we discovered that ERS-related hub genes, such as CASP1 and MAPK14, were positively associated with immune cells, specifically neutrophils. CYBA was positively associated with resting monocytes, whereas NCF1 was negatively associated with resting CD4 + memory T cells. In different cell types, MAPK14 (P38α) is considered a critical regulator of inflammation. Certain subsets of cytokines and chemokines are tightly regulated by p38 signaling in astrocytes (79). CASP1 is known to exert pro-inflammatory effects, which can regulate IL-1β's maturation (80). Ncf1 (p47phox) is essential for direct suppression of CD4+ effector T cells (Teffs) by regulatory T cells (Treg) (81). CYBA (p22phox) has been identified as a novel regulator of monocytes (Mos) and dendritic cells (DCs) differentiation (82). Our findings provide additional evidence that immunity contributes to MDD development. It also verifies our hypothesis that ER stress can modulate the immunological microenvironment of MDD to influence the disease's course.

According to the DSigDB database, we identified fifteen small molecule medications targeting ERSR-DEGs that may be effective in treating MDD, including cinnamaldehyde, estradiol, and ketamine. Trans-Cinnamaldehyde (TCA) has been reported to have significant antidepressant-like effects (83). And supplementation with cinnamaldehyde can induce autophagy and minimize ER stress (84). Estradiol has been identified in previous clinical investigations as a potential treatment or prophylactic for perimenopausal depression (85) and postpartum depression (86). As revealed in a systematic review, estradiol may play a role in depression by regulating the expression of genes that are associated with 5HT neurotransmissions, such as TPH-2, MAO-A and-B, SERT, and 5-HT1A (87). Estradiol, an ERS modulator, has been shown to prevent chondrocyte apoptosis caused by ERS (88). Ketamine is a phencyclidine derivative, which has been approved for treatment-resistant depression (89). Moreover, a recent animal study has shown that ketamine-induced neurotoxicity is associated with an ER stress-dependent apoptotic pathway (90). It has been shown in previous studies that ERS is a potential target for antidepressant therapies (13, 22). The results of our study demonstrate once again that MDD can be treated by targeting genes related to the endoplasmic reticulum.

Our study had a few limitations. First, our research is based on the analysis of data, and further experiments are required to verify our findings. Second, an integrated analysis of both blood samples and brain tissue is required to identify MDD dysfunctions comprehensively, which was not carried out in the current study due to the difficulty in obtaining normal human brain tissue samples. Third, not all patients demonstrate significant ERS due to the heterogeneity of depression. Future research must include subgroup analysis based on the clinical and pathological characteristics of depressed patients. Fourth, the sample size of our study was relatively small, which may have affected our analysis of gene expression in MDD. Although microarray-based bioinformatics analysis can be useful for identifying potential biomarkers of MDD, additional research is necessary to establish the biological significance of ERS.

## Conclusions

Depression affects many people and imposes an enormous economic burden. Therefore, more effort is required to improve its diagnosis and treatment. The heterogeneity, stigma, and lack of effective treatments for depression are significant difficulties. The development of non-invasive validated objective markers not only aids psychiatrists in developing personalized diagnostic and therapeutic strategies for patients with MDD, but also contributes to a more complete understanding of the pathophysiology of depression. Our study was the first to examine comprehensively the biological significance of ERS-related biomarkers and their association with immune infiltrations in MDD. NCF1, MAPK14, CASP1, CYBA, and TNF were identified as MDD biomarkers related to ERS. A putative connection between ERS and the immune system may also play a role in the development of MDD. In addition, regulatory networks upstream and downstream of ERS as well as novel perspective ERS-targeting drugs that may postpone the onset of MDD were investigated. We believe these findings can aid in the development of early diagnostic tools, preventative strategies, and pharmaceutical treatments for MDD.

## Data availability statement

Publicly available datasets were analyzed in this study. The names of the repository/repositories and accession number(s) can be found in the article/Supplementary material.

## Author contributions

JZ conceived and designed this study. SX, YC, XZ, and ZZ collected the data and aided in data analysis. SX, YC, and JZ provided significant suggestions on the methodology. JZ conducted data management and bioinformatics analysis and drafted the manuscript. LY and YL edited and revised the manuscript. All authors read and approved the final manuscript.

## Conflict of interest

The authors declare that the research was conducted in the absence of any commercial or financial relationships that could be construed as a potential conflict of interest.

## Publisher's note

All claims expressed in this article are solely those of the authors and do not necessarily represent those of their affiliated organizations, or those of the publisher, the editors and the reviewers. Any product that may be evaluated in this article, or claim that may be made by its manufacturer, is not guaranteed or endorsed by the publisher.
