# Supplementary material for: Comprehensive analysis of endoplasmic reticulum stress and immune infiltration in major depressive disorder
Source: Front Psychiatry. 2022 Oct 24;13:1008124. doi: 10.3389/fpsyt.2022.1008124 (PMC9638134; doi:10.3389/fpsyt.2022.1008124)
Supplement: Supplementary file 1 [file Data_Sheet_1.zip › fpsyt.2022.1008124 - Table Attached/Table 1.docx]

Supplementary Material

# Supplementary Tables

Supplementary Table 1 The enrichment results of GSEA.

| **Description** | **EnrichmentScore** | **p.adjust** |
| --- | --- | --- |
| GO_ENDOPLASMIC_RETICULUM_LUMEN | 0.305197 | 0.042305 |
| GO_PROTEIN_LOCALIZATION_TO_  ENDOPLASMIC_RETICULUM | -0.46818 | 0.029652 |
| GO_ESTABLISHMENT_OF_PROTEIN_  LOCALIZATION_TO_ENDOPLASMIC_RETICULUM | -0.53932 | 0.029652 |
| GO_SPECIFIC_GRANULE | 0.721262 | 0.029652 |
| GO_TERTIARY_GRANULE | 0.664348 | 0.029652 |
| GO_VESICLE_LUMEN | 0.593184 | 0.029652 |
| GO_SECRETORY_GRANULE_MEMBRANE | 0.553698 | 0.029652 |
| GO_SPECIFIC_GRANULE_MEMBRANE | 0.613563 | 0.029652 |
| GO_MITOCHONDRIAL_GENE_EXPRESSION | -0.51487 | 0.029652 |
| GO_RNA_METHYLATION | -0.58518 | 0.029652 |
| GO_RIBOSOME_BIOGENESIS | -0.48842 | 0.029652 |
| GO_RIBOSOMAL_LARGE_SUBUNIT_BIOGENESIS | -0.60692 | 0.029652 |
| GO_RIBONUCLEOPROTEIN_COMPLEX_BIOGENESIS | -0.4844 | 0.029652 |
| HALLMARK_HEME_METABOLISM | 0.573089 | 0.006207 |
| HALLMARK_IL6_JAK_STAT3_SIGNALING | 0.513163 | 0.006207 |
| HALLMARK_EPITHELIAL_MESENCHYMAL_TRANSITION | 0.4284 | 0.006207 |
| HALLMARK_NOTCH_SIGNALING | 0.532334 | 0.014098 |
| HALLMARK_REACTIVE_OXYGEN_SPECIES_PATHWAY | 0.440569 | 0.020241 |
| HALLMARK_MYC_TARGETS_V2 | -0.4206 | 0.020241 |
| HALLMARK_G2M_CHECKPOINT | -0.36334 | 0.006228 |
| HALLMARK_E2F_TARGETS | -0.44614 | 0.006228 |
| HALLMARK_MYC_TARGETS_V1 | -0.55594 | 0.006228 |
| REACTOME_ACTIVATION_OF_MATRIX_METALLOPROTEINASES | 0.662517 | 0.04469 |
| REACTOME_ERBB2_ACTIVATES_PTK6_SIGNALING | 0.821642 | 0.04469 |
| REACTOME_RHO_GTPASES_ACTIVATE_NADPH_OXIDASES | 0.670314 | 0.04469 |
| REACTOME_INTERFERON_ALPHA_BETA_SIGNALING | 0.518474 | 0.04469 |
| KEGG_LYSOSOME | 0.472818 | 0.04469 |
| WP_VITAMIN_DSENSITIVE_CALCIUM_SIGNALING_IN_DEPRESSION | 0.553012 | 0.04469 |
| REACTOME_ACTIVATION_OF_THE_PRE_REPLICATIVE_COMPLEX | -0.59955 | 0.04469 |
| REACTOME_MITOCHONDRIAL_TRANSLATION | -0.51024 | 0.04469 |
| REACTOME_RRNA_PROCESSING | -0.49315 | 0.04469 |
| KEGG_RIBOSOME | -0.5924 | 0.04469 |
| REACTOME_SRP_DEPENDENT_COTRANSLATIONAL_  PROTEIN_TARGETING_TO_MEMBRANE | -0.56873 | 0.04469 |

Abbreviations: GSEA, Gene set enrichment analysis; GO, gene ontology; KEGG, Kyoto Encyclopedia of Genes and Genomes.

Supplementary Table 2 The enrichment results of GSVA.

| **ID** | **logFC** | **adj.P.Val** |
| --- | --- | --- |
| SA_FAS_SIGNALING | -0.942459735 | 2.03E-33 |
| SA_CASPASE_CASCADE | 0.474954204 | 9.56E-27 |
| SIG_INSULIN_RECEPTOR_PATHWAY_IN_CARDIAC_MYOCYTES | -0.641176996 | 1.02E-24 |
| SA_G1_AND_S_PHASES | -0.420574164 | 9.23E-12 |
| SA_PROGRAMMED_CELL_DEATH | 0.313506247 | 3.63E-07 |
| SIG_CD40PATHWAYMAP | 0.199982268 | 8.09E-06 |
| SIG_CHEMOTAXIS | -0.194758296 | 0.000763 |
| SA_MMP_CYTOKINE_CONNECTION | 0.191350615 | 0.008162 |
| SA_TRKA_RECEPTOR | -0.143985853 | 0.067956 |
| SIG_BCR_SIGNALING_PATHWAY | -0.086131686 | 0.146603 |
| GOBP_CELL_CYCLE | 0.948463494 | 7.19E-56 |
| GOBP_TRANSCRIPTION_BY_RNA_POLYMERASE_III | 1.038765537 | 2.75E-41 |
| GOBP_IMMUNE_SYSTEM_DEVELOPMENT | -0.982920444 | 6.72E-41 |
| GOBP_PROTEIN_MATURATION | 1.020145628 | 7.46E-38 |
| GOBP_REGULATION_OF_CELL_POPULATION_PROLIFERATION | 0.90235761 | 1.50E-36 |
| GOBP_REGULATION_OF_CELL_DIFFERENTIATION | -0.929316711 | 1.63E-29 |
| GOBP_NEUROGENESIS | 0.794445718 | 1.28E-20 |
| GOBP_MAINTENANCE_OF_CELL_NUMBER | 0.767293715 | 7.57E-17 |
| GOBP_DEFENSE_RESPONSE | 0.650680616 | 2.04E-15 |
| GOBP_AMYLOID_PRECURSOR_PROTEIN_METABOLIC_PROCESS | -0.688964532 | 1.03E-13 |
| HALLMARK_EPITHELIAL_MESENCHYMAL_TRANSITION | 0.097930877 | 0.001471 |
| HALLMARK_MYC_TARGETS_V1 | -0.173849585 | 0.020531 |
| HALLMARK_REACTIVE_OXYGEN_SPECIES_PATHWAY | 0.145302859 | 0.032155 |
| HALLMARK_MYOGENESIS | 0.115650555 | 0.032155 |
| HALLMARK_ANGIOGENESIS | 0.165176374 | 0.060022 |
| HALLMARK_MTORC1_SIGNALING | -0.111669682 | 0.060022 |
| HALLMARK_UNFOLDED_PROTEIN_RESPONSE | -0.071892006 | 0.066582 |
| HALLMARK_KRAS_SIGNALING_DN | 0.071696089 | 0.066582 |
| HALLMARK_NOTCH_SIGNALING | 0.100879673 | 0.073772 |
| HALLMARK_CHOLESTEROL_HOMEOSTASIS | 0.091351014 | 0.077612 |

Abbreviations: GSVA, Gene Set Variation Analysis; GO, gene ontology; BP, biological process.
